# Direct endoscopic full-thickness resection for submucosal tumors with an intraluminal growth pattern originating from the muscularis propria layer in the gastric fundus

**DOI:** 10.1186/s12876-020-01215-0

**Published:** 2020-03-12

**Authors:** Jinlong Hu, Nan Ge, Sheng Wang, Jintao Guo, Xiang Liu, Guoxin Wang, Siyu Sun

**Affiliations:** Department of gastroenterolgy, Shengjing Hospital of China Medical University, Shenyang, 110004 China

**Keywords:** Gastric submucosal tumor, Endoscopic full thickness resection, Over the scope clip

## Abstract

**Background and aims:**

Endoscopic full-thickness resection (EFTR) is difficult to perform in a retroflexed fashion in the gastric fundus. The present study aims at exploring whether direct EFTR can be a simple, effective and safe procedure to treat intraluminal-growth submucosal tumors originating from the muscularis propria.

**Methods:**

The patients with intraluminal-growth submucosal tumors originating from the muscularis propria in gastric fundus treated by direct EFTR between 01 January 2017 and 01 September 2018 were retrospectively reviewed. In addition, we analyzed the patients with intraluminal-growth submucosal tumors originating from the muscularis propria in gastric fundus treated by traditional EFTR. The differences in tumor resection time, cost-effectiveness, and complication rate were evaluated.

**Results:**

Forty patients were enrolled in the present study, 20 patients of which were in the direct EFTR group and 20 patients of which were in the traditional EFTR group. En-bloc resections of gastric tumors were successfully performed in all 40 cases. There was no significant difference in the average tumor size of the two groups (24.3 ± 2.9 mm in direct EFTR group verus 24.0 ± 2.6 mm in the traditional group, *p* = 0.731), but significant difference existed in the operative time between two groups (35.0 ± 8.2 min in direct EFTR group verus 130.6 ± 51.9 min in the traditional group, *p*<0.05). No complications, such as postoperative bleeding and perforation, occurred in any groups.

**Conclusions:**

Direct EFTR is a safe, simple and cost-effective procedure for SMTs with an intraluminal growth pattern originating from the muscularis propria layer in the gastric fundus.

## Background

Endoscopic full-thickness resection (EFTR) is applied to resect gastrointestinal submucosal tumors (SMTs) [1]. Compared with surgery and laparoscopy, EFTR could be a more minimally invasive choice for patients and provide the same therapeutic effect for gastric SMTs [2]. However, EFTR is a technical challenging procedure, especially when the lesion is located in the gastric fundus because of retroflexion of the endoscope and specific anatomical features. In order to simplify the procedure of EFTR, loop-mediated, rope-mediated or dental floss traction-assisted EFTR for SMTs in the gastric fundus has been reported [3, 4]. For intraluminal-growth SMTs originating from the muscularis propria in gastric fundus, it can be resected by a more simple method, direct EFTR.

The aim of this study was to evaluate the usefulness of direct EFTR for the resection of intraluminal-growth SMTs originating from the muscularis propria in gastric fundus.

## Methods

### Patients

The patients with intraluminal-growth SMTs originating from the muscularis propria in gastric fundus treated by direct EFTR between 2017/01/01 and 2018/09/01 were retrospectively reviewed; these patients were defined as the trial group. Based on the tumor size and growth pattern, the patients with similar gastric SMTs treated by traditional EFTR were defined as the control group. All the lesions were evaluated by preoperative endoscopic ultrasound (EUS) and computed tomography. Intraluminal growth pattern was defined as: the lesion was of intraluminal growth under endoscopic view and EUS and computed tomography showed that the whole lesion is in the side of gastric cavity.

All the patients signed the informed consent and this study was in accordance with Helsinki Declaration and approved by the ethics committee of Shengjing Hospital of China Medical University.

### Endoscopic equipment and accessories

A standard single-channel gastroscope (EPK-i, Pentax) and transparent cap was used throughout the endoscopic procedure; A snare was used to resect the tumor in the trial group. A triangle-tipped knife and insulated-tip knife (both from Olympus Corporation, Tokyo, Japan) was used for the dissection and resection of tumors in the traditional EFTR group. During the procedure, A hot biopsy forceps (FD-410LR) was used for hemostasis. Metal clips (Olympus Corporation, Tokyo, Japan), and the OTSC system (Ovesco Endoscopy GmbH, Tuebingen, Germany) were used for defect closure.

### Procedures

**Fig. 1:**
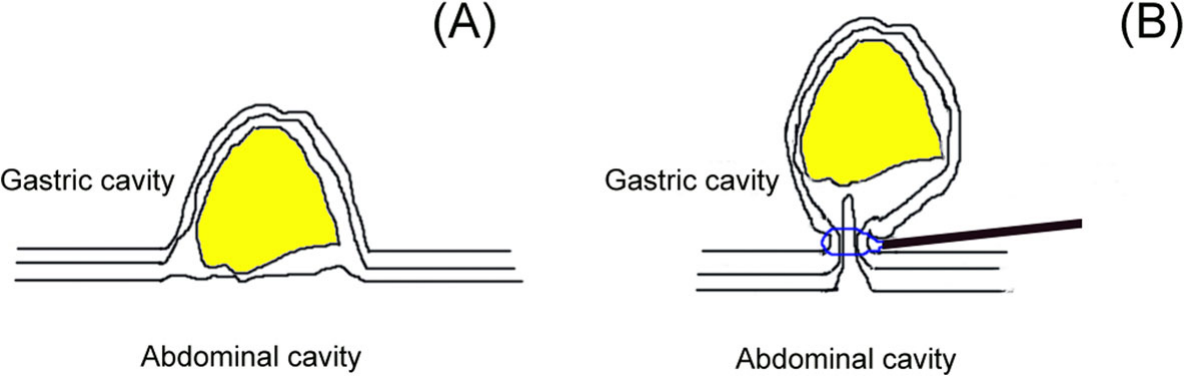
**a** the lesion with intraluminal growth pattern was located in the gastric fundus. **b** the snare was placed at the root of lesion and mild suction was applied to loosen the gastric wall and ensure the complete grasp of the tumor. After the snare was tightened, the tumor presented as a pseudo-polyp

**Fig. 2:**
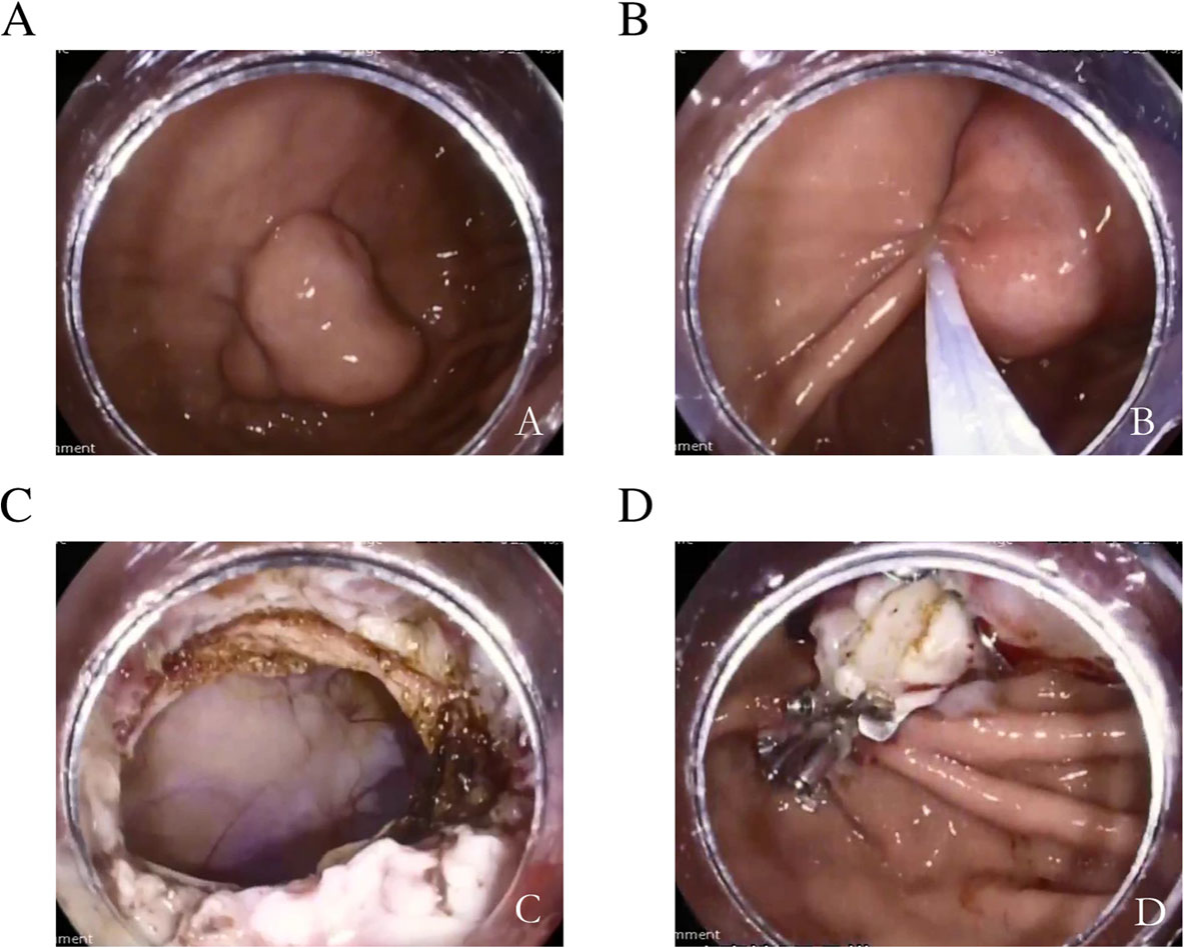
**a** submucosal tumor with intraluminal growth pattern in the gastric fundus; **b**. using a snare to resect the lesion; **c** the iatrogenic perforation after the resection; **d** the perforation was closed by over-the-scope clip and metal clips

All direct EFTR procedures were performed by experienced experts, who can perform traditional EFTR. Direct EFTR was performed as follows (Figs. 1 and 2): First, we aspirated the gastric fluid to empty the stomach. Second, the snare was placed at the root of lesion and mild suction was applied to loosen the gastric wall and ensure the complete grasp of the tumor. After the snare was tightened, the tumor presented as a pseudo-polyp. If there was small amount of tissue in the snare and shaking snare could not continue to tighten the snare, it demonstrated complete grasp of the tumor. Then we resected the tumor and an iatrogenic gastric perforation was created. Third, after resection, electric cautery was used for hemostasis. Fourth, the iatrogenic gastric perforation was closed by metal clips or a metallic clip interrupted suture with an over the scope clip (OTSC).

Traditional EFTR was performed as we previously reported [5].

### Definition of outcomes

The primary outcome measure of this study was en-bloc resection rate. Secondary outcome measures included tumor resection time, cost-effectiveness and adverse events.

### Postoperative treatment and follow-up

The postoperative treatment included fasting, gastric tube decompression as well as routine administration of proton pump inhibitors and antibiotics. Patients were suggested for the endoscopic follow-up at 1,3,6,12 months and annually thereafter to observe recurrence and wound healing.

### Statistical analysis

Statistical analysis was performed with the SPSS software package version 19.0 (SPSS Inc., Chicago, IL, USA).

## Results

In the direct EFTR group, there were 12 males and 8 females, with an average age of (52.6 ± 12.8) years. All the gastric SMTs were intraluminal growth comfirmed by EUS, with an average size of 24.3 ± 2.9 mm. The en-bloc resection rate was 100%. Ten perforation wounds were sealed with over the scope clip (OTSC); ten were closed by OTSC and titanium clips. Operative time ranged from 12 to 47mins, with mean of 35.0 ± 8.2 min. In the traditional EFTR group, there were 10 males and 10 females, with an average age of (54.6 ± 11.8) years. All the gastric SMTs were intraluminal growth comfirmed by EUS, with an average size of 24.0 ± 2.6 mm. The en-bloc resection rate was 100%. 12 perforation wounds were sealed with over the scope clip (OTSC); Eight were closed by OTSC and titanium clips. Operative time ranged from 68 to 240 mins, with mean of 130.6 ± 51.9 min. There was no significant difference between the two groups about tumor size(*p* = 0.731), but the operative time of direct EFTR group was significantly less than that of traditional EFTR group(*p* < 0.05). In both groups, there were no postoperative complications, such as bleeding and perforation (Table 1). Of 40 tumors, there were 39 gastrointestinal stromal tumors and one leiomyoma. Complete resection of all the lesions was done.

**Table 1:** Demographic and clinical characteristics of the direct and traditional groups

Item	Direct EFTR group(*n* = 20)	Traditional EFTR group(*n* = 20)	*P* value
Mean age, year	52.6 ± 12.8	54.6 ± 11.8	*P > 0.05*
Gender (male: female)	12:8	10:10	
Tumor size, mm	24.3 ± 2.9	24.0 ± 2.6	*P > 0.05*
Operative time, minute	35.0 ± 8.2	130.6 ± 51.9	*P < 0.05*
En-bloc resection rate	100%	100%	
Complications			
Postoperative perforation	0	0	
Postoperative bleeding	0	0	

*EFTR* endoscopic full thickness resection

The costs between two groups were different. The cost comparison for the whole procedure was 23,352 ± 512CNY(traditional EFTR) vs 17,033 ± 681CNY(direct EFTR) and there was significant difference(*P* < 0.05).

## Discussion

Gastric SMTs can be exactly diagnosed by EUS [6, 7]. Considering the malignant potential of some SMTs, especially when the tumor size is more than 20 mm, resection is recommened for the lesion. With the development of endoscopic equipment, EFTR has been widely used for gastric SMTs [8, 9].

The SMT located in gastric fundus sometimes is difficult to access, even with the retroflexion of the endoscope, which makes the resection procedure difficult. The traction-assisted EFTR has been reported to improve EFTR procedure in gastric fundus [3, 4]. There were some advantages of traction-assisted EFTR. First, traction can help to expose the tumor boundaries and make the operation filed clear, which can simplify the operation process. In addition, traction can help to quickly locate bleeding point during the procedure, then we can do hemostasis and this method can prevent accidental damage of extravascular vessels. What’s more, traction can help prevent the tumors from falling into abdominal cavity and help to remove the tumor. Although this method has been used for EFTR, EFTR is also a difficult procedure and should be performed by experienced endoscopists.

Usually, the gastric SMTs present as a slightly-protruded lesion. With the growth of SMTs, Some gastric SMTs form an intraluminal growth pattern, like a pseudo-stalk polyp, due to the gravity. For this kind lesion, the tumor is totally in gastric cavity, which can be confirmed by EUS. In our study, we performed direct EFTR for these lesions, like using a mucosa resection for a polyp, which can simply the procedure.

Iatrogenic perforation after EFTR can be sutured by endoscopic equipment, such as over the scope clip (OTSC). The OTSC has shown clinical results over conventional methods. However, the OTSC system also has a limited function in regard to the perforation size. In generally, OTSC can fully suture perforations<20 mm [10]. For a perforation>20 mm, complete closure sometimes can not be achieved by one OTSC, but complete closure can be finished by combining OTSC with metal clips. In our study, all 40 cases were successfully closed. There were several advantages of direct EFTR as follows: first, it can make EFTR more easily to perform and compared with traditional EFTR, it can be done in short time. Second, direct EFTR is cost-effective and we just used a snare to resect the leison. Third, even if there is a bleeding after resection, we can locate the bleeding site easily and do hemostasis. Fourth, this technique can make sure the tumor intact capsule and avoid the damage of the tumor during the dissection.

However, when we used direct EFTR, we should pay attention to some points as follows: first, before resection, we must use EUS to confirm the tumor totally in gastric cavity, otherwise we may cut the tumor and increase the risk. Second, the procedure should be performed by the endoscopic doctor with the ability of hemostasis and closure of iatrogenic perforation. Now this technique can just be used for the tumor with an intraluminal growth pattern. In the future, the retrievable anchor may be used for this technique and pull the tumor back the gastric cavity and resect it by a snare. It should be proved by further studies.

Some limitations are present in our study. First, complete grasp of the tumor was evaluated by the endoscopic doctor’s experience. A more reasonable way should be investigated to judge it by further studies. Second, this is a retrospective and single-center study and less cases were included in the present study. Therefore, a multi-center, prospective and randomized study should be conducted.

## Conclusions

In conclusion, direct EFTR is a safe, simple and cost-effective procedure for SMTs.

with an intraluminal growth pattern originating from the muscularis propria layer in the gastric fundus.

## Data Availability

The dataset supporting the conclusions of this article is available from the corresponding author on reasonable request.

## Abbreviations

EFTR: Endoscopic full-thickness resection
EUS: Endoscopic ultrasound
OTSC: Over the scope clip
SMTs: Submucosal tumors

## References

[1] Wang L, Ren W, Fan CQ (2011). Full-thickness endoscopic resection of nonintracavitary gastric stromal tumors: a novel approach. Surg Endosc.

[2] Huang LY, Cui J, Wu CR (2014). Endoscopic full-thickness resection and laparoscopic surgery for treatment of gastric stromal tumors. World J Gastroenterol.

[3] Shi Q, Li B, Qi ZP (2018). Clinical values of dental floss traction assistance in endoscopic full-thickness resection for submucosal tumors originating from the Muscularis Propria layer in the gastric fundus. J Laparoendosc Adv Surg Tech A.

[4] Lu J, Jiao T, Li Y (2016). Facilitating retroflexed endoscopic full-thickness resection through loop-mediated or rope-mediated countertraction (with videos). Gastrointest Endosc.

[5] Guo J, Liu Z, Sun S (2015). Endoscopic full-thickness resection with defect closure using an over-the-scope clip for gastric subepithelial tumors originating from the muscularis propria. Surg Endosc.

[6] Pesenti C, Bories E, Caillol F (2019). Characterization of subepithelial lesions of the stomach and esophagus by contrast-enhanced EUS: a retrospective study. Endosc Ultrasound.

[7] Antonini F, Delconte G, Fuccio L (2019). EUS-guided tissue sampling with a 20-gauge core biopsy needle for the characterization of gastrointestinal subepithelial lesions: a multicenter study. Endosc Ultrasound.

[8] Zhang Y, Mao XL, Zhou XB (2018). Long-term outcomes of endoscopic resection for small (</= 4.0 cm) gastric gastrointestinal stromal tumors originating from the muscularis propria layer. World J Gastroenterol.

[9] Kappelle WFW, Backes Y, Valk GD (2018). Endoscopic full-thickness resection of gastric and duodenal subepithelial lesions using a new, flat-based over-the-scope clip. Surg Endosc.

[10] Zhang XL, Sun G, Tang P (2013). Endoscopic closure of experimental iatrogenic gastric fundus perforation using over-the-scope clips in a surviving canine model. J Gastroenterol Hepatol.

